# The WHO Classification of Haematolymphoid Tumours

**DOI:** 10.1038/s41375-022-01625-x

**Published:** 2022-06-22

**Authors:** Ian A. Cree

**Affiliations:** International Agency for Research on Cancer (IARC), World Health Organization, 150 Cours Albert Thomas, Lyon, 69372 France

**Keywords:** Cancer, Diagnosis

## Introduction

The World Health Organization (WHO) Classification of Tumours provides a definitive classification of all tumours, worldwide. This is essential to underpin the diagnosis and treatment of cancer, as well as research and education. Without it, clinical trial results could not be compared between countries, research results could not be evaluated collectively and epidemiological studies based on cancer registration would be impossible. It really is the basis of cancer science internationally.

## Historical perspective

The 10th World Health Assembly, the governing body of the WHO, which today comprises 194 countries, directed the WHO to ‘continue work on formulating international definitions of nomenclature and statistical classification’. This rather dry phrasing led to the International Classification of Disease series of coding systems and the WHO Classification of Tumours. Dr Leslie Sobin produced the first edition from 1967 to 1981, and the second over the course of 20 years, from 1982 to 2002. The late Dr Paul Kleihues then joined him to produce the 3rd edition from 2000 to 2005, reducing the time taken to produce the series to just 5 years from the previous 20 years. Unfortunately, the pace did not last: the 4th edition took from 2006 to 2018.

Prior to the 5th edition, we surveyed the readership and were told very clearly that the maximum tolerable update timing was 5 years: quite a challenge. We set about doing this, by converting the classification to a hierarchical taxonomy, entered into a database, and by paying careful attention to process management. The team leading this was another concern: the 4th edition had just four series editors (Drs Ohgaki, Bosman, Lakhani and Jaffe), who chose the editors for each volume, who then chose the authors. However well-intentioned and motivated the series editors, the likelihood of bias is obvious, as is the massive amount of work they took on. It is hardly surprising that the last edition took 12 years to complete. The fact that the 4th edition was so successful is a tribute to their abilities, but this could not continue given the explosion of knowledge that had to be processed for the 5th edition.

In 2017, the new head of the WHO Classification of Tumours, Dr Ian Cree, therefore set up an independent editorial board, the standing members of which are nominated by the major societies of pathology around the world [1–4]. This has since been expanded to include genetics and radiology, reflecting the increasingly multidisciplinary nature of the entire classification [5, 6]. Furthermore, expert members are also appointed to the editorial board for each of the volumes, and these, as well as authors, are selected by a process we know as ‘informed bibliometrics’, which involves looking at who is publishing substantive work in a given area over the last 5 years, informed by where they practice and by the opinion of standing members or, on occasion, international societies. This also meets the requirements of the WHO, which in May 2016 set up a ‘framework for engagement with non-state actors’ to ensure that organisations it dealt with were properly governed and worthy of participation.

The new approach in the 5th edition worked quite well across all blue books completed thus far. The only problem area was the classification of haematolymphoid tumours, which had previously used an ad-hoc ‘clinical advisory committee’ (CAC). Unfortunately, despite the fact that the CAC was a self-appointed group, it had nevertheless been allowed to take over the production of the 4th edition and to produce another 4th edition (revised) version 10 years later—and that volume took 3 years to produce. The CAC has been run by many of the same individuals with few changes for many years, and its selection process for new members is obscure. This contrasts with the WHO Classification of Tumours editorial board. Standing members can serve a maximum of two terms of 3 years each, for a total of 6 years. There was a previously unwritten rule in the WHO Classification of Tumours that experts can only serve as editors for two volumes, unless there are exceptional circumstances, and we have adopted that in writing for the 5th edition, with few exceptions.

The entire WHO classification is now held in a single database organised around an enhanced hierarchical taxonomy, which facilitates changes to the classification during the writing and editorial phases of production, and which mandates a systematic description of the shared characteristics of each tumour type. This also highlights the dearth of evidence in some areas—gaps in knowledge, which we hope will be addressed in future editions [7, 8]. Introductions to each chapter allow editors to identify what has changed from the previous edition and why, as well as giving a detailed explanation of choices made in the classification and their likely impact on diagnosis.

Quality-related issues identified to be addressed in the 5th edition related to the need to improve the quality of figures (an essential aspect, as we have switched to a two-column format with larger illustrations), the use of standardized international units [9, 10], HGNC/HGVS notation of genetics, avoidance of misleading terminology [11] and improved epidemiology. This has gained further importance as we continue to evolve the WHO Blue Books website (https://whobluebooks.iarc.fr), which permits even broader dissemination of the WHO classification and accessibility to it as a worldwide resource.

## The 5th edition classification of haematolymphoid tumours

The haematolymphoid tumours volume had the usual two editorial board meetings, the first in June 2021 and the second in November 2021. As published [3], the first of these meetings set the draft classification (table of contents) to which authorship groups were assigned. In line with the ethos of the 5th edition, these were multidisciplinary groups. When possible, they included hematopathology, genetics, and oncology/clinical haematology experts who have published on the subject within the last 5 years. Furthermore, in this volume, the expert editorial board was carefully selected to include broad multidisciplinary expertise, thereby infusing the effort from the beginning with a range of inputs that reflect current practice. With 420 experts involved in the volume as authors or editors, the range of expertise contributing to this volume is unprecedented. In addition, first, we sought public input on the initial outline (table of contents) of the classification by putting it out for public review and input on the WHO Blue Books website, with FAQs detailing the process followed. Feedback was reviewed by the editorial board, and changes were considered necessary.

The second editorial board considered the written evidence for each tumour type provided by the authorship groups. The scientific debate was to a very high standard and many issues were explored. In addition, a dedicated meeting was organised to receive expert clinical haematology/oncology input on the clinical implications of some of the changes introduced in this edition. The resulting ‘to do’ list took several months to complete and was followed by a final editorial board in a short format in March 2022. At the time of writing, the volume is now undergoing IARC editorial checking prior to technical editing. A beta version is expected to go online in July 2022, which allows feedback from users of the subscription website (https://tumourclassification.iarc.who.int/welcome/) with the publication of the printed book towards the end of 2022, all being well.

The outcome of this effort is another classification in the series that will help move the field forward by being based on a forward-looking multidisciplinary effort grounded in genetic advances, with an eye on worldwide applicability. An overview of the classification and its salient features are provided in the two excellent companion manuscripts, which cover the classification of myeloid and histiocytic/dendritic neoplasms [12] and the classification of lymphoid neoplasms [13].

## Disclaimer

The content of this article represents the personal views of the author and does not represent the views of the author’s employers and associated institutions. Where the author is identified as personnel of the International Agency for Research on Cancer/World Health Organization, the author alone is responsible for the views expressed in this article, and does not necessarily represent the decisions, policy or views of the International Agency for Research on Cancer/World Health Organization.
